# Myrrh Oil-Based Nanoemulsion Loaded with Curcumin and Insulin: Development, Characterization, and Evaluation of Enhanced Antibacterial and Diabetic Wound-Healing Activity

**DOI:** 10.3390/pharmaceutics18030369

**Published:** 2026-03-16

**Authors:** Ayman Salama, Mona Qushawy, Nehal Elsherbiny, Helal F. Hetta, Saleh F. Alqifari, Mohamed A. Safwat, Wael M. Elsaed, Mahmoud Elsabahy, Yasmin N. Ramadan, Ghareb M. Soliman

**Affiliations:** 1 Department of Pharmaceutics, Faculty of Pharmacy, University of Tabuk, Tabuk 71491, Saudi Arabia; agrawan@ut.edu.sa (A.S.); mqushawy@ut.edu.sa (M.Q.); 2 Department of Pharmaceutical Chemistry, Faculty of Pharmacy, University of Tabuk, Tabuk 71491, Saudi Arabia; nelsherbiny@ut.edu.sa; 3 Division of Microbiology, Immunology and Biotechnology, Department of Natural Products and Alternative Medicine, Faculty of Pharmacy, University of Tabuk, Tabuk 71491, Saudi Arabia; hhussen@ut.edu.sa; 4 Department of Pharmacy Practice, Faculty of Pharmacy, University of Tabuk, Tabuk 71491, Saudi Arabia; salqifari@ut.edu.sa; 5 Department of Pharmaceutics, Faculty of Pharmacy, Qena University (Formerly South Valley University), Qena 83523, Egypt; safwat_mohamad@svu.edu.eg; 6 Department of Human Anatomy and Embryology, Faculty of Medicine, Mansoura University, Mansoura 35516, Egypt; wael.zaarina@riyadh.edu.sa; 7 Preclinical Department, College of Medicine and Dentistry, Riyadh Elm University, Riyadh 12734, Saudi Arabia; 8 Badr University in Cairo Research Center, Badr University in Cairo, Badr City, Cairo 11829, Egypt; mahmoud.elsabahy@buc.edu.eg; 9 Department of Pharmaceutics, Faculty of Pharmacy, Assiut University, Assiut 71515, Egypt; 10 Department of Microbiology and Immunology, Faculty of Pharmacy, Assiut University, Assiut 71515, Egypt; yasmine_mohamed@pharm.aun.edu.eg

**Keywords:** curcumin, insulin, myrrh oil, nanoemulsion, diabetic wounds, nanoemulgel

## Abstract

**Background/Objectives**: Curcumin (CUR) has shown promising potential as a wound-healing agent for diabetic wounds; however, its efficacy is hindered by poor aqueous solubility and limited skin permeability. To overcome these limitations, CUR was loaded into myrrh oil-based nanoemulsions (NEs). **Methods**: The NEs were optimized using a three-factor two-level D-optimal mixture design, and characterized for droplet size, polydispersity index, and zeta potential. The optimized NE was subjected to various stability testing and incorporated into a gel base containing insulin (INS) to form CUR-INS nanoemulgel (CUR-INS-NEG). The antibacterial efficacy of CUR-INS-NEG was tested against various bacterial strains, while its wound-healing effects were evaluated in a diabetic rat wound model. **Results**: The surfactant/co-surfactant concentration had a greater influence on the NE properties than the oil and aqueous phase concentrations. The optimal NE had a droplet size of 155.2 ± 0.8 nm, a polydispersity index of 0.28, and a zeta potential of −31.4 ± 0.8 mV. It demonstrated sustained drug release, with further release control upon incorporation into the gel base. CUR-INS-NEG demonstrated higher in vitro antibacterial efficacy compared with blank NEG, CUR gel, and INS gel. It also showed 2- and 4-fold reduction in the MIC against *S. aureus* and *E. coli*, respectively, compared with CUR gel. In a diabetic wound model, CUR-INS-NEG outperformed both CUR gel and INS gel by enhancing anti-inflammatory and antioxidant effects, as well as collagen deposition and endothelial cell proliferation. **Conclusions**: The CUR-INS-NEG emerges as an effective system for diabetic wound management, delivering complementary anti-inflammatory, antioxidant, and tissue-regenerative effects of myrrh oil, CUR, and INS.

## 1. Introduction

Diabetes mellitus (DM) is one of the most challenging diseases due to its profound adverse effects on almost every system in the body. This metabolic disorder, primarily characterized by chronic hyperglycemia, leads to macrovascular manifestations, such as coronary heart disease, cardiomyopathy, arrhythmias, cerebrovascular disease, and peripheral artery disease. Its microvascular complications include retinopathy, nephropathy, and neuropathy, all of which significantly impair the patient’s quality of life. The global DM prevalence in 2019 was estimated to be 9.3% (463 million people), which is projected to rise to 10.2% (578 million) by 2030 and 10.9% (700 million) by 2045 [1]. Global DM economic burden is expected to increase from US $1.3 trillion in 2015 to $2.2 trillion by 2030. This includes direct expenses of treatment and indirect ones due to limited productivity, disability, and premature mortality [2]. Moreover, DM also causes social burdens, including psychosocial distress, anxiety, stigma, and poor quality of life [3]. Diabetic wounds are among the most challenging conditions to manage and treat due to several factors, including poor blood circulation, neuropathy, immune dysregulation, impaired angiogenesis, and high blood sugar levels [4]. These factors contribute substantially to the impaired wound-healing process and increased risk of infection. Previous studies have shown that 50–60% of diabetic foot ulcers become infected, with moderate to severe cases leading to amputation in about 20% of cases [5].

Wound healing is a biologically complex process that restores skin integrity and repairs damaged tissue. This process is dynamic, progressive, and occurs in four overlapping stages: hemostasis, inflammation, proliferation, and remodeling [6]. Impaired or delayed healing of diabetic wounds is a serious clinical problem that imposes a substantial health burden [7]. The International Working Group on the Diabetic Foot/Infectious Diseases Society of America for diabetes-related foot infections indicated that the treatment of diabetic wounds should follow a multidisciplinary and evidence-based approach. This includes rapid diagnosis of infection, treatment with selective antibiotics, local wound care, and hyperglycemia control [8]. These strategies suffer from several limitations, including limited efficacy, persistent inflammation, oxidative stress, biofilm formation, impaired angiogenesis, and high recurrence rates (40% within one year). This often leads to delayed wound healing [7]. Innovative drug delivery systems such as nanoparticle-based formulations have shown great potential to overcome these limitations through controlled, targeted, and stimuli-responsive drug delivery. These systems have achieved improved drug bioavailability, reduced dosing frequency, limited systemic toxicity, and improved wound healing [9,10,11].

Insulin (INS) is a peptide hormone secreted by the β-cells of the pancreas in response to elevated blood glucose levels [12]. It has long been known for its powerful wound-healing properties [13]. Topical INS administration has been found to be safe and effective for the treatment of various animal and human wounds, such as diabetic ulcers, pressure sores, and corneal defects, leading to reduced healing time [13,14]. Topical INS is much safer than systemic INS, resulting in improved wound healing without causing hypoglycemia and hypokalemia in diabetic and non-diabetic patients [15]. Moreover, topical INS offers the advantages of rapid granulation, upregulation of local growth factors, and increased efficacy in refractory wounds [16]. However, topical INS suffers from several drawbacks, including limited penetration through the skin due to its large hydrophilic structure, rapid enzymatic degradation within the chronic wound environment, and short half-life. These limitations warrant the development of advanced formulations to improve its topical stability and therapeutic efficacy in diabetic wounds.

Curcumin (CUR) is a naturally occurring bioactive compound derived from the rhizomes of turmeric (*Curcuma longa* L.). It has been widely used in traditional medicine due to its potent anti-inflammatory, antibacterial, and antioxidant properties [17,18,19]. CUR topical administration has been extensively studied for wound healing, leading to enhanced tissue repair [20,21]. CUR also possesses antibacterial properties against both Gram-positive and Gram-negative bacteria, which enhance its wound-healing properties by limiting wound infection [22]. However, CUR clinical application in wound healing is limited by poor aqueous solubility, limited skin penetration, and low bioavailability.

Myrrh oil is an essential oil derived from *Commiphora myrrha* resin. It has shown wound-healing properties due to its contents of sesquiterpenes and furanoeudesma-1,3-diene [23]. These compounds also have anti-inflammatory, antimicrobial, and analgesic effects, which support tissue repair and improve wound-healing properties [24,25].

Nanoemulsions (NEs) are transparent, kinetically stable systems that are formed by high-energy emulsification and are stabilized by a mixture of surfactant and co-surfactant. They are composed of nano-sized internal phase droplets (10–100 nm) dispersed within the external phase [26,27]. They have shown superior advantages for topical drug delivery in wound-healing applications [28]. The presence of oil and aqueous phases in NEs enables the simultaneous encapsulation of lipophilic drugs, such as CUR in the oil phase, and hydrophilic drugs, like INS in the aqueous phase, forming a single stable delivery system [29,30]. NEs have shown a dramatic increase in the aqueous solubility of hydrophobic drugs such as CUR and protected sensitive drugs from degradation [30]. The nanoscale size of NE facilitates deep skin penetration and targeted drug delivery to the wound bed [31]. Indeed, NEs have shown a strong potential as drug delivery platforms for diabetic wounds [32]. For instance, INS-loaded NE incorporated into Aloe vera gel demonstrated 75% wound contraction in 15 days compared with 15% for the control [29]. In another study, CUR NEs demonstrated faster closure and re-epithelialization (~96.47%) of diabetic wounds compared with the control [33]. These results clearly demonstrate the potential of NEs as a promising delivery system with enhanced wound-healing properties and controlled drug release, thereby improving patient compliance. The literature reveals a notable lack of studies combining the clinical benefits of CUR, INS, and myrrh oil into a single formulation to harness their potential complementary effects in wound healing.

The aim of this study was to develop a triple NE formulation using myrrh oil as the oil phase to encapsulate CUR, with INS solubilized into the external aqueous phase. This delivery system combines the antimicrobial, anti-inflammatory, antioxidant, and wound-healing properties of myrrh oil, CUR, and INS. The NE was incorporated into a gel base to facilitate its topical application, increase residence time on wounds, and enhance its efficacy, which eventually results in improved patient compliance. The developed systems were characterized using several techniques, and the formulation that produced the best attributes was evaluated in vivo in a streptozotocin-induced diabetic wound model.

## 2. Materials and Methods

### 2.1. Materials

CUR, Pluronic^®^ F127 (PL-F127), Tween 80 (TW80), and low-molecular-weight chitosan (CS) were purchased from Sigma-Aldrich (St. Louis, MO, USA). Transcutol and oleic acid were purchased from Central Drug House Ltd. (New Delhi, India). Myrrh oil, lemon oil, and tea tree oil were obtained from Hemani General Trading LLC (Karachi, Pakistan). Commercial INS (Human Actrapid^®^ Insulin, 100 IU/mL) was obtained from Novo Nordisk (Bagsværd, Denmark). Sesame oil was obtained from Asaggaf Pharma Holyland (Riyadh, Saudi Arabia). Nutrient broth, Mueller–Hinton agar, resazurin, and brain heart infusion broth were obtained from Thermo Fisher Scientific (Waltham, MA, USA).

### 2.2. Solubility Studies of CUR

CUR solubility in different oils (oleic acid, lemon oil, tea tree oil, sesame oil, and myrrh oil), surfactant (TW80), and co-surfactant (Transcutol) was determined according to previously reported procedures with slight modifications [34]. Briefly, an excess of CUR was added to 2 mL of the given excipient in a small, closed vial. The vials were continuously stirred on a magnetic stirrer at 400 RPM and room temperature for 72 h. Aliquots from the vials were centrifuged at 10,000 RPM for 10 min, and the supernatant was separated and diluted with ethanol. CUR concentration was determined using a UV–Vis spectrophotometer at λ_max_ of 422 nm (Cary 5000 UV–Vis Spectrophotometer, Santa Clara, CA, USA).

### 2.3. Construction of Pseudo-Ternary Phase Diagram

Six mixtures of the surfactant (TW80) and co-surfactant (Transcutol) were prepared (Smix) in different TW80/Transcutol volume ratios (1:0, 1:0.35, 1:0.5, 1:1, 1:2, and 1:3). For the construction of each phase diagram, the oil, and a given Smix ratio were mixed thoroughly in different oil/Smix volume ratios (1:9, 1:7, 1:5, 1:4, 1:3, 1:2, 1:1, and 2:1). The boundaries of phases formed in the phase diagram were determined. The pseudo-ternary phase diagrams were developed using the aqueous phase titration method, where the aqueous phase (5–95% of total volume) was slowly added to each oil/Smix mixture [35]. After each addition, visual observation was made and recorded. The following categories were assigned to the formed system [27]:

1. Transparent and easily flowable: Nanoemulsion.
2. Transparent gel: Nanoemulsion gel.
3. Milky or cloudy: Emulsion.
4. Milky gel: Emulgel.

### 2.4. Preparation of CUR-Loaded NEs

CUR-loaded NEs were prepared using the ultrasonication method according to previously published procedures with minor modifications [27,36]. Briefly, CUR was dissolved in the oil at a concentration of 0.2% *w*/*v*. The Smix was mixed with water to form the aqueous phase. The oil was added slowly to the aqueous phase and mixed using a magnetic stirrer at 600 RPM for 15 min to form a coarse emulsion [27]. The resulting emulsions were homogenized using a high shear homogenizer (NanoGenizer-30K; Genizer, Irvine, CA, USA) by six homogenization cycles at a pressure of 10,000 psi [37]. The mixtures were further sonicated for 10 min using a digital sonifier (Qsonica, LLC, Newtown, CT, USA) with intermittent sonication (30 s pulse ON and 30 s pulse OFF) to avoid overheating, at a power of 500 W and an amplitude of 25%, until a clear, transparent solution was obtained. During the sonication process, the samples were kept under an ice bath to prevent a temperature increase.

### 2.5. Optimization of CUR-Loaded NE Formulations

This study employed a three-factor two-level D-optimal mixture design. The effects of three independent variables, namely the oil percentage (X_1_), Smix% (X_2_), and water percentage (X_3_), on the response variables, namely the droplet size (Y_1_), polydispersity index (PDI) (Y_2_), zeta potential (Y_3_), and drug content% (Y_4_), were evaluated (Table 1). A design matrix of the 12-point D-optimal experiment was constructed using Design-Expert software version 11 (Stat-Ease, Minneapolis, MN, USA). Each design was assessed separately to assess the influence of the composition of each variable on the four responses [38]. The statistical validity of the generated polynomial equations was established based on the ANOVA provision in the software. The models were evaluated in terms of statistically significant coefficients and *R*^2^ values [35].

### 2.6. Selection of the Optimized NE Formulation

The optimization aimed to minimize particle size (Y_1_) and PDI (Y_2_) while maximizing the absolute zeta potential (Y_3_) and drug content percentage (Y_4_). The software applied a desirability function to combine all responses, searching for the formulation with the highest overall desirability index.

### 2.7. Measurement of Droplet Size, Polydispersity Index, and Zeta Potential

The NE droplet size, PDI, and zeta potential were evaluated at 25 °C by the dynamic light scattering (DLS) technique using a Malvern ZetaSizer Nano Series ZS 90 (Malvern Instruments, Malvern, Worcestershire, UK). Aliquots of the samples were diluted 10-fold with distilled water and measured in triplicate at room temperature [27].

### 2.8. Drug Content Measurement

The CUR content of various NE formulations was determined using previously published procedures with minor modifications [27,39]. Briefly, an aliquot of each formulation (0.2 mL) was mixed with 20 mL of ethanol. The mixture was then shaken for 30 min in a shaking incubator at 50 RPM and 37 ± 0.5 °C. The CUR content of an aliquot was determined spectrophotometrically at a *λ*_max_ of 422 nm. The CUR content was presented as a percentage of the CUR amount initially used in the NE preparation.

### 2.9. Physical Stability Testing

The optimized NE formulation (NE12) was subjected to different stress stability tests.

#### 2.9.1. Centrifugation Study

An aliquot was centrifuged at 5000 RPM for 30 min on a Hermle Z326 centrifuge (HERMLE Labortechnik, Wehingen, Germany) and observed for phase separation, creaming, or cracking [27,35].

#### 2.9.2. Heating–Cooling Cycles

Aliquots were subjected to six cycles between 4 °C and 40 °C, with storage for at least 48 h at each temperature [27].

#### 2.9.3. Freeze–Thaw Cycles

Aliquots were subjected to three freeze–thaw cycles at temperatures between −21 °C and 25 °C, with storage at each temperature for at least 48 h [27].

#### 2.9.4. Dilution Test

An aliquot (1 mL) was diluted with 10 mL of distilled water and observed for phase inversion [40].

### 2.10. pH Measurement

The pH of an aliquot was measured at 25 °C using an AD8000 pH meter (Adwa, Hungary).

### 2.11. Transmission Electron Microscopy (TEM) Measurements

The morphology and size of the optimized NE formulation (NE12) were characterized using a transmission electron microscope (JEOL GEM-1010, JEOL Ltd., Tokyo, Japan). An aliquot of the formulation was diluted with distilled water, placed on a carbon-coated copper grid, and left to dry at room temperature. The sample was negatively stained using aqueous phosphotungstic acid solution (1% *w*/*v*). The grid was then allowed to dry overnight at room temperature and was observed using the TEM machine operating at 80 kV.

### 2.12. Fourier Transform Infrared (FT-IR) Studies

The spectra of NE12, CUR, TW80, Transcutol, and myrrh oil were recorded on a Digilab Spectrum Excalibur FT-IR spectrometer (Digilab, Randolph, MA, USA) equipped with an attenuated total reflectance (ATR) accessory. Data collection was performed in the range of 4000–400 cm^−1^ at room temperature at a resolution of 4 cm^−1^.

### 2.13. Differential Scanning Calorimetry (DSC) Studies

DSC thermograms of the same FT-IR samples were recorded using a TA Discovery DSC 25 (TA Instruments, Waters LLC, New Castle, DE, USA). The samples (3–5 mg) were tightly sealed in aluminum pans and heated in the range of 30 to 350 °C at a scanning rate of 10 °C/min under a nitrogen gas flow of 25 mL/min. An empty pan was used as a reference.

### 2.14. Preparation of Different Gel Formulations

To prepare 10 g of CUR nanoemulgel containing INS (CUR-INS-NEG), 5 mL of NE12 was added dropwise to 5 mL of 1% *w*/*v* chitosan (CS) solution in 0.5% *v*/*v* acetic acid under continuous magnetic stirring at 500 RPM for 15 min. Subsequently, 0.1 mL of INS (Human Actrapid^®^ Insulin, 100 IU/mL, Novo Nordisk, Bagsværd, Denmark) was added, and stirring was continued for an additional 15 min. The resulting solution was stored overnight at 4 °C. Next, 2 g of PL-F127 was added to the cold solution slowly over a period of approximately 5–10 min with gentle stirring on a magnetic stirrer, and the mixture was left overnight at 4 °C to ensure complete dissolution of PL-F127 [41]. Blank nanoemulgel (B-NEG) was prepared by identical procedures, except that it contained neither CUR nor INS. Control PL-F127 gel was prepared using the previously published cold method [42]. Control CS-PL-F127 gel was prepared by adding 2 g of PL-F127 to 10 mL of cold CS solution (0.5% *w*/*v* in 0.5% *v*/*v* acetic acid) slowly over a period of about 5–10 min with gentle stirring on a magnetic stirrer, and the mixture was left overnight at 4 °C to ensure complete dissolution of PL-F127. Control CUR-G (without NE) was prepared by dissolving the calculated CUR amount in 0.5 mL of DMSO, followed by mixing with 10 mL of cold CS-PL-F127 solution, and the rest of the procedures were the same as described above. Control INS-G (without NE) was prepared by mixing the calculated volume of INS with 10 mL of cold CS-PL-F127 solution, and the rest of the procedures were the same as described above. The final concentrations were 0.5% for CS, 20% for PL-F127, 0.2% for CUR, and 1 IU/g gel for INS based on the type of gel. The INS concentration used in this study was selected based on previously reported topical INS wound-healing studies demonstrating enhanced re-epithelialization and angiogenesis without inducing systemic hypoglycemia [16,43,44]. The applied dose falls within the range commonly used in experimental diabetic wound models.

### 2.15. Characterization of the Prepared Gels

#### 2.15.1. Measurement of pH

The pH of the prepared gels was measured at 25 °C using an AD8000 pH meter (Adwa, Hungary).

#### 2.15.2. Measurement of Gelation Temperature (T_sol-gel_)

The gelation temperature was measured according to previously reported procedures [45]. Briefly, 10 mL of each formulation was placed into a beaker, and a thermometer was immersed in the sample solution. The sample was heated at a constant rate of ~2 °C/min under continuous stirring by a small magnetic bar at 200 RPM. The temperature at which the magnetic bar stopped rotating due to sample gelation was taken as the gelation temperature.

#### 2.15.3. Measurement of Spreadability

A 0.5 g sample was placed on a pre-marked circle on a glass slide, covered with a second slide, and compressed with a 500 g load for 1 min. The resulting spread diameter was taken as the spreadability value [46].

#### 2.15.4. Viscosity Measurement

The viscosity was measured using an AMETEK Brookfield Cap 2000+ viscometer (Middleboro, MA, USA) operating at 37 ± 0.3 °C. The samples were placed in a water bath at 37 °C until complete gelation was obtained. The viscosity was measured at 37 °C using spindle 5 rotating at 10 RPM and a shear rate of 34 s^−1^.

#### 2.15.5. Storage Stability Studies

This study was carried out according to the “Stability testing of biotechnological/biological products” guidelines based on the International Conference on Harmonization (ICH), Q5C [47,48]. Freshly prepared CUR-INS-NEG was stored at 5 °C in tightly closed containers for three months. Subsequently, samples were visually examined and tested for INS content, pH, viscosity, homogeneity, droplet size, PDI, and zeta potential. The INS content of the gel was determined by dispersing 0.2 g of the formulation in 10 mL of distilled water, and the mixture was shaken in a water bath shaker for 30 min at 50 RPM and 25 ± 0.5 °C. The INS concentration of an aliquot was determined from its spectrophotometric absorbance at 271 nm [47]. The droplet size, PDI, and zeta potential were determined by dispersing 0.5 g of the gel sample in 10 mL of distilled water, followed by magnetic stirring at 400 RPM for 20 min, and the measurements were taken as described under Section 2.7.

### 2.16. In Vitro Drug Release Studies

The release of CUR from formulation NE12 was compared with that of CUR NE12 NEG and CUR solution in methanol using a standard semipermeable cellophane membrane. The release medium was phosphate buffer (50 mL, pH 7.4) supplemented with 1% *v*/*v* Tween 80 to maintain sink conditions. A piece of cellophane membrane was used to close one end of a glass tube that was open at both ends, and it was secured with a rubber band. The tested formulation (1 mL equivalent to 1 mg CUR) was placed over the membrane in the tube. This study was performed at 37 ± 0.2 °C and 50 RPM using a shaking water bath. To generate the release curves, samples (5 mL) were taken from the release medium at different time intervals and replaced with an equal volume of release medium maintained at 37 °C. CUR concentration in the samples was measured spectrophotometrically at 422 nm. The cumulative amount of CUR released was calculated and plotted against time.

Regarding the INS release, the investigated formulations were INS aqueous solution, INS NE12, and INS NE12 NEG. A sample from each formulation (equivalent to 5 units INS) was placed over the dialysis membrane, which was placed in 10 mL of phosphate buffer, pH 7.4, as a release medium maintained at 37 °C and agitated at 50 RPM. Aliquots (2 mL) were taken from the release medium at 15, 30, 45, 60, 90, and 120 min and analyzed for INS concentration spectrophotometrically at 271 nm [47].

### 2.17. Antibacterial Studies

#### 2.17.1. Screening of Antibacterial Activity

The antibacterial efficacies of blank nanoemulgel (B-NEG), free curcumin gel (CUR-G), free insulin gel (INS-G), and CUR nanoemulgel containing insulin (CUR-INS-NEG) were evaluated using the agar cup diffusion technique [49]. The tested bacterial strains were *Staphylococcus aureus* (ATCC 6538), *Escherichia coli* (ATCC 8739), *Klebsiella pneumoniae* (clinical isolate), *Pseudomonas aeruginosa* (ATCC 90274), and *Salmonella typhimurium* (ATCC 14028) [50]. The bacterial cultures were incubated overnight at 37 °C on Mueller–Hinton agar to obtain fresh colonies. A new bacterial suspension was then adjusted to a 0.5 McFarland standard and evenly spread over the agar surface using sterile swabs. A cork borer was used to create wells in the agar, having a 1 cm diameter. An aliquot of the tested preparation (100 μL) was added to each well. Inhibition zones diameters were measured following an overnight incubation at 37 °C.

#### 2.17.2. Determination of Minimum Inhibitory Concentrations (MICs)

MICs were determined using a microdilution assay in 96-well microtiter plates according to previously described procedures with minor modifications [50]. Briefly, an aliquot of nutrient broth (100 μL) was transferred to each well of the microtiter plates. Subsequently, the tested preparations (100 μL of each) were added to the wells of the first vertical row of the plates. Gentamicin (0.025 mg/mL) (Garamycin^®^ injection, Memphis Pharmaceutical and Chemical Industries, Cairo, Egypt) was used as a positive control. Two-fold serial dilutions were made, and 100 μL from the last well was discarded. Bacterial suspensions (1.5 × 10^5^ CFU/mL, 100 μL) from *S. aureus* and *E. coli* were added to the wells. The plates were incubated overnight at 37 °C. Resazurin dye (10 μL) was added to each well and incubated for a further 4 h at 37 °C to determine the MICs.

#### 2.17.3. Anti-Biofilm Assay

This was performed following previously reported procedures with minor modifications using *S. aureus* and *E. coli* isolates as representatives of Gram-positive and Gram-negative bacteria, respectively [51]. Briefly, the bacterial strains were cultured in brain heart infusion broth and incubated overnight at 37 °C. Samples of the tested preparations at concentrations below the MIC were added to 96-well microtiter plates and mixed with diluted bacterial cultures (1.5 × 10^8^ CFU/mL). Negative controls were used as wells containing only broth, while positive controls were wells having untreated bacterial cultures. Following incubation for 24 h at 37 °C, the wells were gently emptied and rinsed 2–5 times with 200 μL of sterile normal saline to eliminate non-adherent bacteria. The attached biofilms were dried at 65 °C for 30 min, followed by staining with 200 μL of a 0.1% crystal violet solution. Unbound dye was eliminated by rinsing each well five times with 200 μL of normal saline. After air drying, the retained stain in the biofilms was solubilized with 33% glacial acetic acid, and the absorbance was recorded at 570 nm using a SPECTROstar Nano microplate reader (BMG Labtech, Germany) after 15–30 min. The percentage of biofilm inhibition was calculated using the following equation:

$$Percentage\;\left(\%\right)\;inhibition=\;\frac{Absorbance\;of\;positive\;control-Absorbance\;of\;treated\;well}{Absorbance\;of\;positive\;control}\;\times \;100$$

Biofilm inhibition was categorized into three levels: good inhibition for values exceeding 50%, weak inhibition for values between 0% and 50%, and biofilm enhancement for values below 0% [49,50].

### 2.18. In Vivo Wound-Healing Studies

The animals (forty Sprague Dawley rats, weighing 200–250 g) were maintained under standard laboratory conditions of humidity, temperature, and lighting, with free access to a standard pelleted diet and water. This study was approved by the Institutional Animal Ethical Committee (approval number P.S.V.U-2/1/2025, Faculty of Pharmacy, Qena University, Egypt). The procedures complied with the ARRIVE guidelines and were carried out in accordance with the *National Research Council Guide for the Care and Use of Laboratory Animals*, 8th edition (National Academies Press, Washington, DC, USA).

#### 2.18.1. Induction of Diabetes Mellitus (DM)

The animals were acclimated for one week and randomly divided into a control group (eight animals) and a model group, in which thirty-two animals were injected intraperitoneally with a freshly prepared streptozotocin solution (STZ, 50 mg/kg). Blood glucose was assessed using a digital glucometer three days after the STZ injection, and blood glucose levels over 250 mg/dL were indicative of successful DM induction.

#### 2.18.2. Wound Incision

One week following DM induction, the rats were anesthetized intraperitoneally with ketamine at a dosage of 90 mg/kg and xylazine at 10 mg/kg. An electrical hair clipper was used to clip the animal’s dorsal fur, followed by skin disinfection using 70% ethanol. A full-thickness round wound excision was then created. The wounded diabetic animals were randomly assigned to four groups, each of 8 animals. The first group (DM + B-NEG) received B-NEG. The second group (DM + CUR-G) received free CUR gel. The third group (DM + INS-G) received free INS gel. The fourth group (DM + CUR-INS-NEG) received CUR-INS-NEG. The treatments were topically applied daily for 3 weeks in all groups. The wound-healing capacity was evaluated by measuring the wound diameter and area. The following equation was used for calculating the wound closure rate (WCR):

$$WCR=\;\frac{iWA-fWA}{iWA}\times 100\;\;$$

where iWA is the initial wound area at day 0, and fWA is the final wound area at day 20 [52].

#### 2.18.3. Sample Collection

Animals were sacrificed at the end of the experimental period, and skin tissue samples were collected. Each sample was divided into two parts. The first part was preserved in buffered formalin for histopathological and immunohistochemical analyses, while the second part was stored at −80 °C for further biochemical and molecular analyses.

#### 2.18.4. Histopathological Evaluation

Preserved skin specimens were fixed in paraffin and cut into sections having a thickness of 4 μm, each coded for analysis. One set was stained using hematoxylin and eosin (H&E) to study general tissue architecture. To assess the deposition of collagen in the dermal layer, another set was stained with Masson’s trichrome. Histological alterations were identified and captured using a light microscope equipped with a digital camera (Olympus^®^, Tokyo, Japan).

#### 2.18.5. Assessment of Oxidative Stress

An enzyme-linked immunosorbent assay (ELISA) kit was used to measure the concentration of nuclear factor erythroid 2-related factor 2 (Nrf-2, BT LAB, Shanghai, China) in skin tissue homogenate following the manufacturer’s procedures. Malondialdehyde (MDA) and reduced glutathione (GSH) levels were measured spectrophotometrically using commercially available kits (Biodiganostics, Cairo, Egypt) according to the manufacturer’s procedures.

#### 2.18.6. Assessment of Inflammation

The ELISA method was used for measuring the concentration of nuclear factor kappa-light-chain-enhancer of activated B cells (NF-κB) and tumor necrosis factor alpha (TNF-α) (CUSABIO, Wuhan, Hubei Province, China), and interleukin-6 (IL-6) (R&D Systems, Minneapolis, MN, USA) in skin tissue homogenate using commercially available kits and following the manufacturer’s protocol.

#### 2.18.7. Immunohistochemistry for Transforming Growth Factor-β (TGF-β) and Vascular Endothelial Growth Factor (VEGF)

Skin sections were deparaffinized and incubated overnight with primary antibodies for TGF-β and VEGF (ABclonal Co., Woburn, MA, USA) at a dilution of 1:500. The immunoreaction was then detected by incubating the slides with horseradish peroxidase-conjugated secondary antibodies. This was followed by the addition of 3,3′-diaminobenzidine tetrahydrochloride (Genemed, Biotechnologies Inc., San Francisco, CA, USA) for visualization. Finally, Mayer’s hematoxylin was used for counterstaining the slides to be examined. The stained area was measured using ImageJ software version K 1.45.

### 2.19. Statistical Analysis

All experiments were performed in triplicate at least, and data are presented as the mean ± standard error (SE) for the in vivo experiments and as the mean ± standard deviation (SD) for all other measurements. Normality testing using the Shapiro–Wilk test confirmed that the data were normally distributed. A statistical comparison was performed using an analysis of variance (ANOVA) test followed by a post hoc Tukey’s test (GraphPad Prism software version 8.3.0, San Diego, CA, USA). Statistical significance was set at *p*-value < 0.05.

## 3. Results and Discussion

### 3.1. Determination of CUR Solubility

Among the test oils, myrrh oil demonstrated the highest CUR solubility of 11.89 ± 0.25 mg/mL, confirming its ability to form NE with high drug loading. Solubility in other oils was 2.38 ± 0.19 mg/mL for lemon oil, 6.27 ± 0.17 mg/mL for oleic acid, 7.54 ± 0.11 mg/mL for sesame oil, and 8.25 ± 0.05 mg/mL for tea tree oil. Accordingly, myrrh oil was selected as the NE oil phase. Additionally, TW80 and Transcutol, used as surfactant and co-surfactant, demonstrated high CUR solubility of 50.42 ± 0.75 mg/mL and 62.73 ± 0.91 mg/mL, respectively, confirming their suitability for formulating NE with high CUR loading.

### 3.2. Construction of Pseudo-Ternary Phase Diagrams

**Figure 1:**
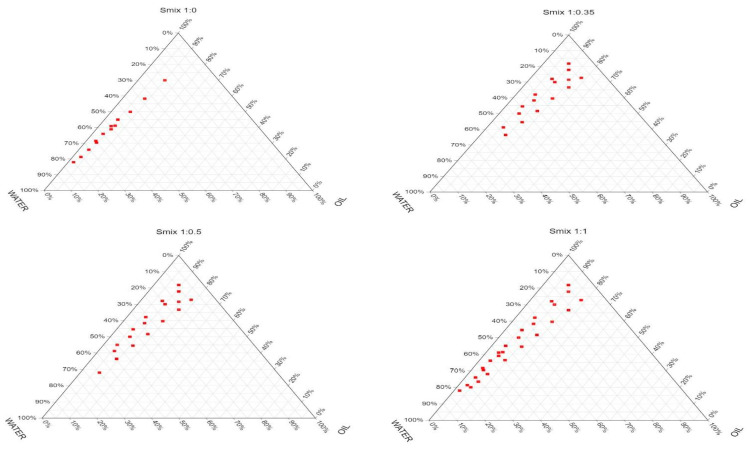

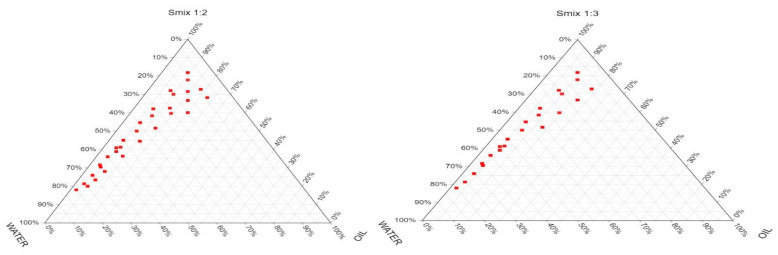
Pseudo-ternary phase diagrams of various NE compositions of myrrh oil, water, and Smix at different Smix ratios, with the NE area shown as closed circles.

Six Smix ratios (1:0, 1:0.35, 1:0.5, 1:1, 1:2, and 1:3) were evaluated to study their effects on NE formation, given the critical role of surfactant and co-surfactant relative concentrations in determining the size and position of the NE region [27,35]. The shaded area in Figure 1 represents the translucent O/W NE region, while the rest of the phase diagram represents the conventional emulsion. The NE area was small when TW80 was used alone (Smix 1:0), indicating its inability to sufficiently reduce the interfacial tension between the oil and water phases and stabilize the NE. TW80 bulky hydrophilic head groups created a rigid layer around the oil droplets, leading to coalescence [53]. The area of the isotropic NE region increased with increasing the co-surfactant concentration up to a Smix ratio of 1:1, which was also reported in previous findings [27,54]. When the Smix ratio was increased to 1:2, the NE area also increased, although the extent of increase was less pronounced compared to the earlier ratios. Previous reports demonstrated that single surfactants cannot substantially reduce the interfacial energy required for the formation of NE, necessitating the addition of a co-surfactant. The co-surfactant role is to reduce the interfacial tension and increase the fluidity at the oil/water interface [53,55]. The increase in co-surfactant concentration generally results in decreasing the interfacial tension until a certain point, after which the interfacial tension increases [55]. Upon further increasing the co-surfactant to Smix ratio to 1:3, the NE area was insignificantly reduced, indicating that the maximum reduction in interfacial tension was achieved, which is consistent with previous studies [56]. The Smix ratio of 1:2 was chosen for the optimization study, as it provided the widest NE area, as determined by the cut-and-weigh method.

### 3.3. Model Fitting of Responses

Model fitting for each response was conducted using Design-Expert software, utilizing the D-optimal mixture design. Sequential *p*-values and adjusted and predicted *R*^2^ values were used to assess the model’s suitability. The quadratic model was found to be the optimal fit for droplet size (Y_1_), with a *p*-value of 0.0032, an adjusted *R*^2^ of 0.9902, and a predicted *R*^2^ of 0.9689 (Table S1). This demonstrates the high accuracy and predictability of this model. The special cubic model had the best fit for PDI (Y_2_), yielding a *p*-value of 0.0330, an adjusted *R*^2^ of 0.8083, and a predicted *R*^2^ of 0.2816, indicating a moderate fit. The linear model was chosen for zeta potential (Y_3_), demonstrating a significant agreement between the observed and predicted values. This was indicated by a *p*-value < 0.0001, an adjusted *R*^2^ of 0.9440, and a predicted *R*^2^ of 0.9071. Similarly, the linear model was the most appropriate for drug content% (Y_4_). It has a *p*-value < 0.0001, an adjusted *R*^2^ of 0.9375, and a predicted *R*^2^ of 0.8951, which confirms its strong predictive reliability. The chosen models demonstrated statistical significance and were appropriate for explaining the relationship between formulation factors and the observed responses. The selected range of independent variables was narrow (10–15% for the oil, 40–50% for the Smix, and 40–50% for water) to make sure it is within the NE region, which was observed in Figure 1 at the Smix ratio 1:2 (Table 1). These ranges ensure the stability of the obtained formulations and their confinement within the NE region of the phase diagram.

### 3.4. Effect of Formulation Factors on the NE Droplet Size

**Figure 2:**
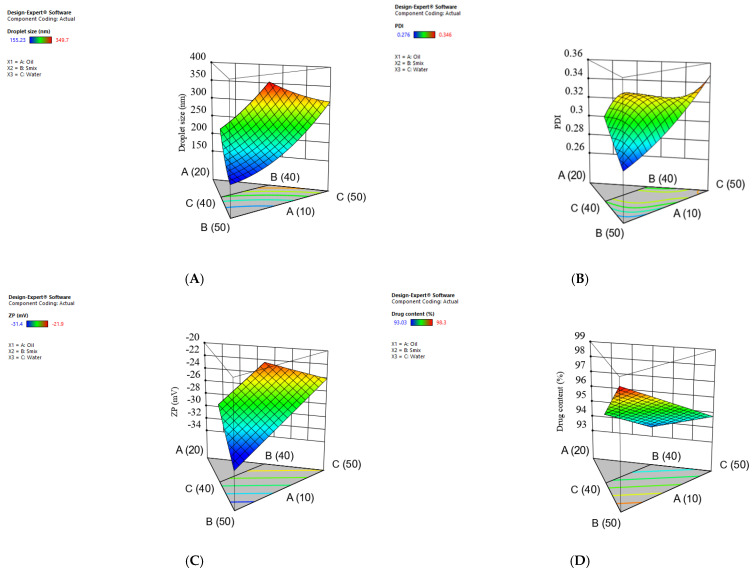
Three−dimensional surface plots for the effect of formulation factors on (**A**) droplet size, (**B**) PDI, (**C**) ZP, and (**D**) drug content%.

The 3D surface response plot shows that increasing the oil concentration led to a progressive increase in droplet size (Figure 2A). This is likely attributed to the higher viscosity and larger internal phase volume, which require more surfactant to reduce the interfacial tension efficiently [57]. In contrast, increasing the Smix content significantly reduced the droplet size. The higher Smix content lowers the interfacial tension and reduces the free energy required to deform the droplets, resulting in smaller droplets. Additionally, Smix may form a protective layer around the oil droplets, which prevents coalescence. These observations are consistent with previous studies [58]. A higher water concentration led to a slight increase in the droplet size. High water content dilutes the surfactant packing around the oil droplets, which may facilitate their coalescence.

Table S2 shows that the quadratic model was highly significant (F = 224.26, *p* < 0.0001). This indicates a pronounced effect of the mixture components on the droplet size. The linear mixture term showed the most significant contribution (F = 537.68, *p* < 0.0001). The interactions AB (F = 9.98, *p* = 0.0196) and BC (F = 31.12, *p* = 0.0014) were also statistically significant. The quadratic model demonstrated exceptional adequacy, as evidenced by an *R*^2^ value of 0.9947, a C.V.% of 2.76, and an adequate precision of 43.85.

### 3.5. Effect of Formulation Factors on the PDI

The 3D surface plot (Figure 2B) shows that increasing the Smix concentration led to reduced PDI values, suggesting a uniform droplet size distribution. This is likely due to the adequate availability of surfactants, which stabilize the dispersed oil droplets. In contrast, the increased oil concentration generally resulted in an increased PDI, likely due to an insufficient surfactant concentration leading to decreased interfacial stabilization and formation of heterogeneous or larger droplets. The increase in water concentration resulted in a minor elevation in PDI, probably due to the dilution effects, which limit surfactant coverage around the oil droplets [59]. As shown in Table S2, the special cubic model for PDI was statistically significant (F = 8.73, *p* = 0.0155), demonstrating that the NE ingredients substantially affected the response. The linear mixture term was the primary contributor (F = 14.21, *p* = 0.0087), while the higher-order interaction ABC also demonstrated a significant effect (F = 8.52, *p* = 0.0330). The model exhibited high adequacy, as shown by *R*^2^ = 0.9129, C.V.% = 3, and an adequate precision of 10.0925.

### 3.6. Effect of Formulation Factors on the Zeta Potential (ZP)

ZP is a crucial criterion that significantly impacts the physical stability of NE through electrostatic repulsion, which prevents droplet aggregation, coalescence, or flocculation [53]. The 3D surface plot shows that the increase in Smix concentration led to an increase in the negativity of ZP values (Figure 2C). This may be related to the increased concentration of the Smix at the oil–water interface. Despite the non-ionic nature of TW80 and Transcutol, their adsorption at the oil–water interface may increase the adsorption of hydroxyl ions, increasing the negativity of the zeta potential [53]. In contrast, an increased oil concentration resulted in a reduction in the ZP negativity, probably due to the decreased surfactant concentration relative to oil, leading to reduced charge density at the interface [60]. Increasing the water fraction resulted in a slight decrease in the magnitude of the negative zeta potential, likely due to dilution and decreased adsorption of surfactants on the oil droplet surfaces.

Table S2 shows that the linear model for ZP was highly significant (F = 93.74, *p* < 0.0001), demonstrating a substantial influence of the NE components on the ZP. The model shows strong reliability, as evidenced by *R*^2^ = 0.9542, C.V.% = 2.82, and an adequate precision of 27.12.

### 3.7. Effect of Formulation Factors on the Drug Content%

The CUR content of the NE refers to the percentage of measured CUR amount in the NE relative to the theoretical CUR content. The 3D surface response plot (Figure 2D) shows that increasing Smix concentration led to increased drug content%. This is likely due to the improved drug solubility by the surfactant/co-surfactant mixture [61]. Higher oil concentrations led to a minor reduction in drug content, which may be due to the formation of larger oil droplets that may restrict uniform drug distribution [62]. Increasing the water concentration resulted in a moderate decrease in drug content, which may be attributed to the dilution effect and the reduction in surfactant concentration. This may have limited the retention of the drug within the oil droplets. As shown in Table S2, the linear model demonstrated high significance (F = 83.56, *p* < 0.0001), indicating a substantial impact of the NE components. The model demonstrated strong adequacy as evidenced by an *R*^2^ of 0.9489, C.V.% of 0.3852, and an adequate precision of 26.11.

### 3.8. Selection of the Optimized Formulation

The optimized NE formulation was determined using a desirability function aimed at minimizing both droplet size and PDI, while maximizing the absolute zeta potential and drug content% to ensure NE stability and high drug loading. The identified optimal formulation contained 10% oil, 50% Smix, and 40% water. This formulation had a predicted droplet size of 150.26 nm, a PDI of 0.28, a zeta potential of −32.77 mV, and a drug content of 98.47% (Figure S1). This was accompanied by a high overall desirability value of 0.988. The experimental values obtained for the optimized formulation (NE12) were 155.2 ± 0.8 nm, 0.28, −31.4 ± 0.8 mV, and 98.3 ± 0.6% for the droplet size, PDI, zeta potential, and drug content%, respectively. These values are very close to the predicted responses, which confirms the accuracy of the optimization process.

### 3.9. NE Physical Stability Testing

NEs are thermodynamically unstable but kinetically stable systems. They are formed at specific ratios of oil, surfactant, and water and show no phase separation, creaming, or cracking over extended periods of time [35,63]. The optimized formulation (NE12) passed all the investigated stress tests. These included centrifugation, heating–cooling cycles, and freeze–thaw cycles. Moreover, it showed no signs of Ostwald ripening. Based on these results, this formulation was selected for further investigations due to its potential as an effective delivery system of CUR. This formulation was readily diluted 10-fold with distilled water and demonstrated no signs of phase inversion. This confirms its classification as an O/W NE [27].

### 3.10. pH Measurement

The pH of NE12 was 6.53 ± 0.01 at 25 °C, confirming its suitability for topical application and thereby limiting its potential for skin irritation.

### 3.11. TEM Studies

**Figure 3:**
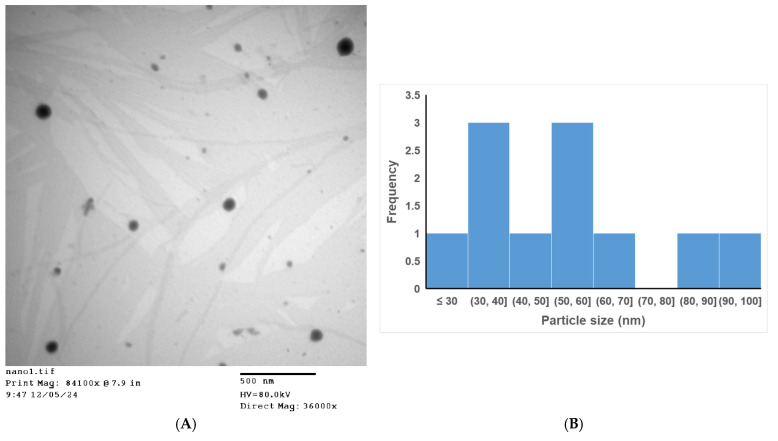
(**A**) TEM photomicrograph of the optimized CUR NE formulation (NE12). (**B**) Histogram of size distribution of NE12 obtained from TEM imaging (*n* = 11).

Figure 3A shows that the NE droplets appear as spherical dark particles with defined boundaries. The particles were discrete, free from aggregation, and had a fairly uniform size distribution. The droplet size obtained from the TEM image ranged from 28.7 to 96.5 nm (mean = 54.57 nm, SD = 21.62 nm). This size distribution indicates a moderate heterogeneity in particle size rather than monodispersed particles (Figure 3B). Previous studies showed that the heterogeneity of NE size distribution was influenced by several factors, including formulation and processing variables that affect the oil droplets’ breakup, coalescence, and stabilization. For instance, high shear homogenization was shown to produce a broad size distribution due to repetitive droplet breakup [64]. Process and formulation parameters, such as vortexing time, surfactant/co-surfactant ratio, and oil phase composition, were also shown to affect the NE droplet size and size distribution [65]. The size obtained from TEM imaging was much smaller than that observed from DLS measurements (155.23 ± 0.8 nm, Table 2). This is commonly observed in the literature and is usually attributed to the dried nature of the TEM samples. In contrast, DLS samples are measured in solution, which explains their larger size due to sample hydration [27,66].

### 3.12. FT-IR Studies

**Figure 4:**
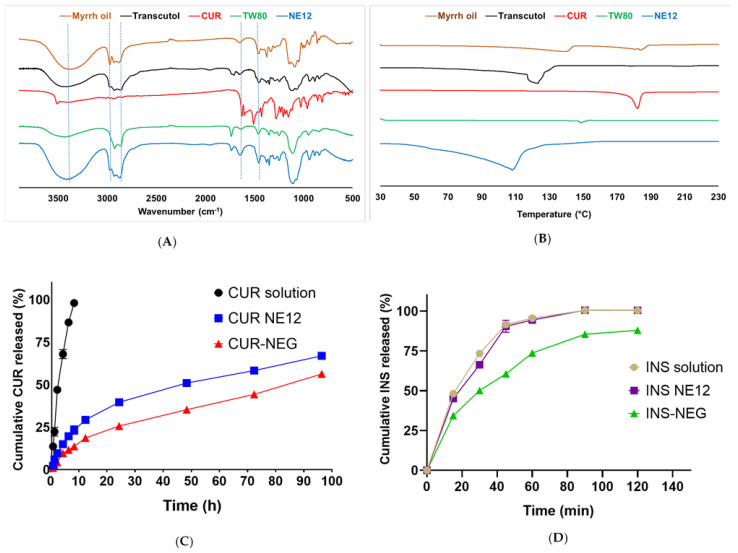
(**A**) FT−IR spectra of myrrh oil, Transcutol, CUR, TW80, and CUR optimized NE12. (**B**) DSC thermograms of myrrh oil, Transcutol, CUR, TW80, and CUR optimized NE12, (**C**) In vitro release profiles of CUR from its solution, NE12, and NE12 nanoemulgel at 37 °C in phosphate buffer pH 7.4. (**D**) In vitro INS release profiles from its aqueous solution, NE12, and NE12 nanoemulgel at 37 °C in phosphate buffer pH 7.4.

Figure 4A shows that the CUR spectrum displays an absorption band at around 3510 cm^−1^, attributed to the stretching vibrations of phenolic and alcoholic hydroxyl groups. A sharp band is shown at 1630 cm^−1^, ascribed to the overlapping stretching vibrations of alkenes (C=C) and carbonyl (C=O) groups [67]. Another sharp and intense absorption band appears at 1510 cm^−1^, which can be attributed to a combination of vibrational modes including carbonyl stretching vibrations, in-plane bending of aliphatic groups (δ CC-C and δ CC=O), and in-plane bending of aromatic C-H bonds (δ CC-H) associated with both keto and enol forms of CUR [68]. The Transcutol spectrum exhibits a band at 3450 cm^−1^, attributed to the strain vibrations of the hydroxyl groups. The bands shown at 2970 cm^−1^ and 2920 cm^−1^ are due to the vibration of C-H bonds of the alkyl groups [69]. The spectrum of myrrh oil exhibits a broad band at 3380 cm^−1^, attributed to the O-H group stretching. The bands at 2971 and 2930 cm^−1^ can be attributed to alkyl C-H groups stretching. The bands at 1735 cm^−1^ and 1450 cm^−1^ are characteristic of ester C=O group stretching and C=C group stretching, respectively [70]. The spectrum of TW80 shows an absorption band at 3440 cm^−1^ due to the stretching vibration of O-H groups. The bands at 2920 and 2860 cm^−1^ are due to the stretching vibrations of alkyl C-H groups. The band at 1730 cm^−1^ is attributed to the ester C=O group stretching [71]. CUR NE12 shows absorption bands at 3417 cm^−1^ due to O-H groups stretching. The absorption bands at 2972, 2925, and 2876 cm^−1^ are characteristic of C-H stretching vibrations from aliphatic hydrocarbon chains. The band at 1735 cm^−1^ is characteristic of ester-C=O-group stretching. Upon comparison of the different spectra, it can be concluded that the observed absorption peaks in the NE12 spectrum are consistent with those of its individual components. This confirms the absence of chemical interactions among the NE constituents, as such interactions typically cause shifts, broadening, or disappearance of specific absorption bands in the FT-IR spectrum [72].

### 3.13. DSC Studies

Thermal changes such as peak shifts, the disappearance of characteristic peaks, enthalpy changes, or the appearance of new exo/endothermic peaks, together with FT-IR band changes, provide stronger evidence for assessing drug–excipient incompatibility than either technique alone [73]. The CUR thermogram shows a sharp endothermic peak at 181.99 °C due to its melting (Figure 4B) [74]. The myrrh oil thermogram shows two endothermic peaks at approximately 140.62 °C and 184 °C, likely due to evaporation and/or thermal decomposition. The Transcutol thermogram shows a broad endothermic peak at around 126.7 °C, probably due to evaporation/volatilization or decomposition-related events [75]. The thermogram of TW80 shows a small endothermic peak at 148.8 °C, which may be attributed to its flash point. Previous studies reported this flash point at around 115 °C [76]. This difference may be due to different experimental conditions, such as the heating rate, sample mass, or atmosphere [77]. The thermogram of the optimized NE12 formulation demonstrates a broad endothermic peak centered around 107.93 °C, probably due to water loss. The CUR peak in the NE12 thermogram disappeared, likely due to a transformation from a crystalline to an amorphous state, resulting from the drug solubility in the NE oil phase. This finding is consistent with results observed in previous studies [74,75].

### 3.14. Characterization of the Prepared Gels

The pH values ranged from 6.01 ± 0.09 to 6.94 ± 0.03, confirming the minimal irritation potential of these gels (Table 3). The pH slightly decreased with the addition of 0.5% CS as it was solubilized in acetic acid. The gels showed T_sol-gel_ around 35 °C, ensuring their gelation at body temperature. The temperature sensitivity of PL-F127 gels is advantageous, where the preparation is liquid at room temperature, providing ease of application for wounded areas where minimal friction and rubbing are desirable to reduce pain, protect fragile new tissue, and lower infection risks [78]. Following application, it is converted to gel at body temperature, providing improved residence time and sustained drug release [79]. The addition of CS to this formulation further enhances the residence time at the wounded area through its mucoadhesive properties, thereby increasing drug efficacy [41]. The viscosity ranged from 122 ± 1.73 to 226 ± 6.24 P based on the preparation. There was a notable increase in the PL-F127 gel viscosity with the addition of 0.5% CS, which may be attributed to the increased molecular entanglement and hydrogen bonding within the gel matrix [80]. The spreadability was inversely related to the viscosity due to its effect on reducing the formulation’s ease of flow under applied shear. CUR-INS-NEG had a spreadability of 47.9 ± 0.6 mm, indicating good ease of application. Previous studies confirmed that topical gels with a spreadability of 50–70 mm show good spreadability for skin application [81].

### 3.15. Storage Stability of CUR-INS-NEG

Stability studies are crucial to verify the ability of the prepared formulations to retain their initial drug content within acceptable limits, while preserving their important physicochemical properties [82]. Samples from CUR-INS-NEG were tested for several parameters after storage at 5 °C for 3 months (Table 4). There was no change in the preparation color, homogeneity, pH, and droplet size at the end of the study period. The observed droplet size (~20 nm) is much smaller than that of the optimal formulation NE12 (~155 nm). However, the size from this measurement may not represent the NE droplets only, as other components of the system, such as PL-F127, may also form micelles upon dilution of the NEG, making it difficult to compare with the droplet size of NE12 before incorporation into the NEG. The preparation viscosity increased from 219 ± 9.0 P to 289 ± 1.73 P, which may be due to some water evaporation from the preparation, making it more viscous. INS content slightly decreased from 98.8 ± 0.36% to 94.8 ± 0.31%, which was within the acceptable 10% change limit for low-stability substances [47,48]. The PDI slightly changed from 0.29 ± 0.005 to 0.32 ± 0.007, indicating that the size distribution of the NE droplets was not adversely affected by storage. The absolute zeta potential value slightly decreased from 46.66 ± 3.72 mV to 41.66 ± 0.11 mV. It is noteworthy that the zeta potential value of NE12 changed from negative (−31.4 ± 0.8 mV, Table 2) to positive (46.66 ± 3.72 mV, Table 4) upon incorporation into the gel, which is likely due to the presence of the positively charged chitosan in the gel. This large, positive zeta potential is an interesting attribute of the NEG due to its positive effects on system stabilization through electrostatic repulsion and therapeutic outcome through mucoadhesion. The results from this storage stability study confirm the stability of CUR-INS-NEG and its ability to retain its INS content and essential physicochemical properties within the acceptable limits.

### 3.16. In Vitro Drug Release Studies

Figure 4C shows that the control CUR solution in methanol had complete release (98.0 ± 2.0%) after 8 h, confirming that no barriers hindered the drug release through the dialysis membrane. CUR incorporation in NE12 markedly slowed its release, with only 23.33 ± 2.52% released after 8 h. The total release from this formulation after 96 h was 67.0 ± 2.0%, confirming the sustained drug release. The formulation of hydrophobic drugs into NEs has been previously reported to slow their release rate due to the high affinity of the drug for the NE oil phase, which reduces partitioning into the external aqueous release medium and leads to sustained release [27]. CUR NE incorporation in the gel base further decreased its release rate, with 13.67 ± 1.15% and 56.33 ± 1.15% being released after 8 h and 96 h, respectively. This is likely due to the increased viscosity of the gel and the additional diffusion barrier it imposes on the movement of the drug, which slows the diffusion of the drug from the oil phase to the release medium [83]. After 8 h and 96 h, the differences between the three tested samples were statistically significant (*p* < 0.05).

Unlike CUR, the control INS aqueous solution rapidly diffused out of the dialysis membrane, resulting in 100% release in approximately 90 min (Figure 4D). Similarly, INS NE12 and INS NEG exhibited fast release as indicated by 100.42 ± 0.42% and 88.01 ± 0.21% release after 120 min, respectively. The cumulative released INS amount from the NEG after 120 min was significantly lower than that from the INS solution and INS NE12, likely due to the diffusion barrier imposed by the hydrogel. The rapid INS release from its NE12 compared with CUR is likely attributed to the presence of the drug in the NE external aqueous phase and its high aqueous solubility, which facilitate its diffusion into the aqueous release medium. Previous studies demonstrated similar observations for INS NEs [84,85]. The observed rapid INS release and sustained CUR release represent a rational strategy for wound healing. Rapid INS availability promotes early wound repair by enhancing keratinocyte and fibroblast proliferation and accelerating re-epithelialization. Moreover, sustained CUR release helps maintain anti-inflammatory and antioxidant activity and may also support tissue repair processes, thereby facilitating tissue remodeling and preventing prolonged inflammation [86].

### 3.17. In Vitro Antibacterial Studies

#### 3.17.1. Screening of Antibacterial Activity

**Figure 5:**
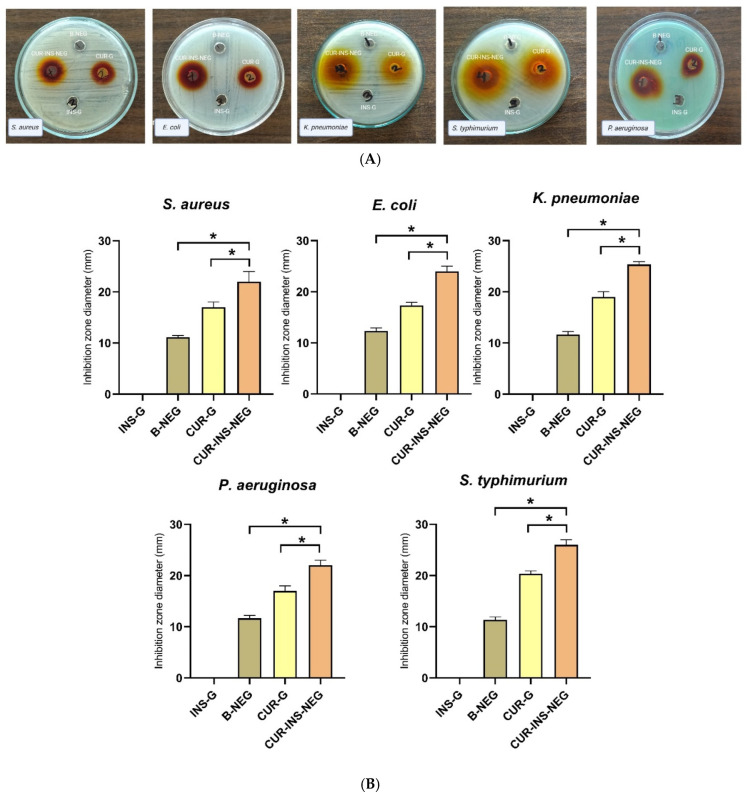
Screening of antibacterial activity of various formulations against selected Gram-positive and Gram-negative bacterial strains. (**A**) Photomicrographs of the plates showing the inhibition zones. (**B**) Average of inhibition zone diameters for various formulations. * Denotes a significant difference (*p* < 0.0001).

Figure 5 shows that INS-G did not exhibit antibacterial activity against any of the tested bacteria. In contrast, B-NEG showed mild antibacterial activity, which is probably attributed to its myrrh oil content. This formulation had inhibition zones ranging from 11.1 ± 0.2 mm to 12.3 ± 0.5 mm. Myrrh oil’s antibacterial activity is due to several active constituents, including terpenes and phenolic compounds [87]. CUR-G exhibited antibacterial activity against all the tested bacterial strains. It had inhibition zones ranging from 17.0 ± 1.0 mm against *S. aureus* to 20.33 ± 0.58 mm against *Salmonella typhimurium*. Interestingly, CUR-INS-NEG demonstrated a significantly higher inhibition zone compared with both B-NEG and CUR-G against all the tested strains (*p* < 0.0001).

#### 3.17.2. MIC Determination

INS-G showed no antibacterial activity. B-NEG demonstrated MIC values of 50 mg/mL and 25 mg/mL against *S. aureus* and *E. coli*, respectively, confirming its mild antibacterial activity. In contrast, CUR-G showed a much lower MIC value of 0.125 mg/mL against both *S. aureus* and *E. coli*. The CUR-INS-NEG demonstrated MICs of 0.063 and 0.031 mg/mL *S. aureus* and *E. coli*, respectively. This represents around a 2- and 4-fold reduction in the MIC against *S. aureus* and *E. coli*, respectively, compared with CUR-G.

#### 3.17.3. Anti-Biofilm Studies

Bacterial biofilms form structured microbial communities within a protective extracellular matrix, which firmly adheres to the wound site. This increases the chronicity of diabetic wounds. Additionally, these biofilms result in prolonged inflammation, impaired fibroblast and keratinocyte functions, and limited granulation tissue formation. These effects lead to poor wound healing and increase risks of complications such as amputation [88]. The anti-biofilm activity of the tested formulation was evaluated against *S. aureus* and *E. coli* as representatives of Gram-positive and Gram-negative bacteria, respectively. For *S. aureus*, the CUR-INS-NEG achieved an inhibition rate of 97.3%, notably higher than the 92.2% and 83.5% inhibition rates observed for CUR-G and B-NEG, respectively. Similarly, the CUR-INS-NEG had a remarkably high inhibition rate of 96.1% against *E. coli*. This is notably higher than the rates of 92.6% and 78.9% achieved for the CUR-G and B-NEG, respectively, against the same strain.

Collectively, these antibacterial studies confirm the superior efficacy of CUR-INS-NEG compared with either CUR-G or B-NEG alone. This is likely due to complementary effects between CUR and myrrh oil. CUR induces reactive oxygen species (ROS)-mediated damage to bacterial cell membranes. This may enhance the ability of myrrh oil sesquiterpenes (e.g., furanoeudesma-1,3-diene) to disrupt the bacterial cell walls and efflux pumps. This complementary activity may have resulted in enhanced inhibition zones, lowered MICs, and improved anti-biofilm activity. Additionally, the NE small droplet size (155.23 ± 0.8 nm) may have enhanced the drug’s aqueous solubility, stability, and ability to penetrate the bacterial biofilm, leading to superior cellular uptake and sustained release compared to the free drug. Previous studies have shown similar findings, where CUR olive oil NE, combined with the antibiotic ceftazidime, has demonstrated synergistic effects leading to a significant reduction in MIC against *K. pneumoniae* resistant clinical isolates [89]. CUR-cinnamon essential oil NE demonstrated significantly lower MIC against *Mycobacterium smegmatis* and *M. tuberculosis* compared with either component alone [90].

### 3.18. In Vivo Studies

#### 3.18.1. Effect of Treatments on Wound Healing

**Figure 6:**
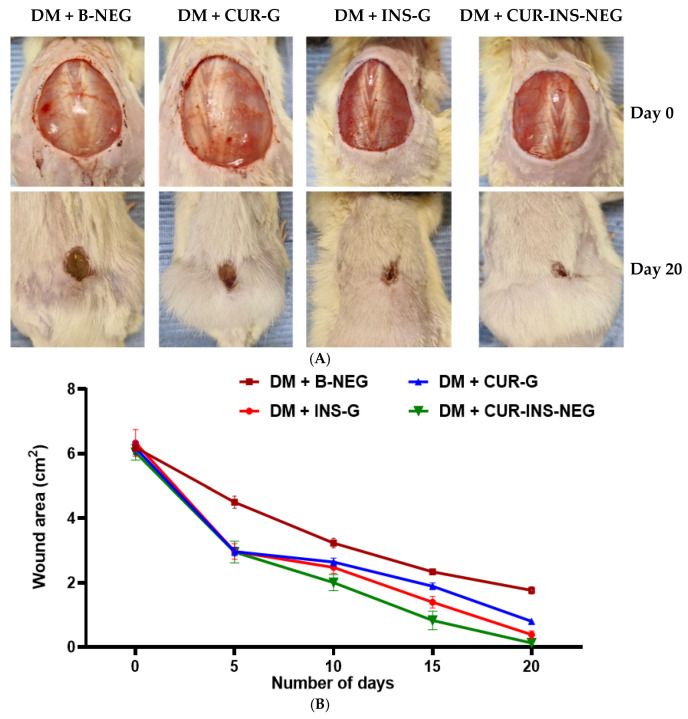
(**A**): Photomicrograph of the wounded area in diabetic rats from the four experimental groups at days 0 and 20. (**B**): Wound area measured over the experimental time at days 0, 5, 10, 15, and 20.

Figure 6 shows that topical application of CUR-INS-NEG resulted in an increased rate of wound healing as indicated by the smallest wound area after treatment for 20 days compared to the B-NEG, CUR-G, and INS-G-treated groups. Specifically, after 20 days, the wound areas were 1.76 ± 0.26 cm^2^, 0.80 ± 0.19 cm^2^, 0.39 ± 0.12 cm^2^, and 0.13 ± 0.10 cm^2^ for the B-NEG, CUR-G, INS-G, and CUR-INS-NEG groups, respectively. All the differences were statistically significant (*p* < 0.05) except those between the INS-G group and the groups treated with either CUR-G or CUR-INS-NEG. The CUR nanoformulations’ ability to improve wound healing was recently reviewed [91]. Moreover, topical INS therapy accelerates wound contraction and re-epithelialization with a marked reduction in healing time [13]. Interestingly, INS has been reported to catalyze curcumin-mediated wound healing in an in vitro model of gingival repair [92].

#### 3.18.2. Effect of Treatments on Wound Oxidative Stress

**Figure 7:**
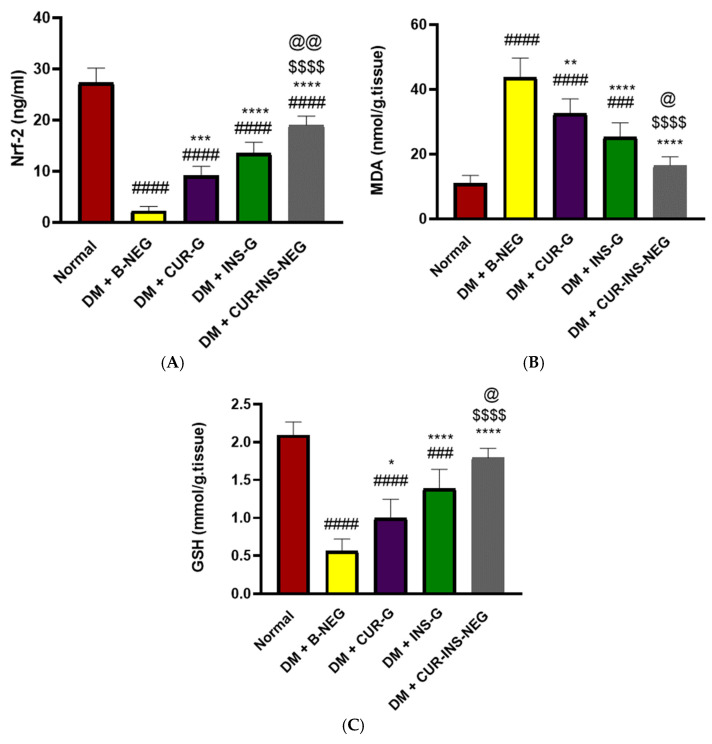
Effect of topical diabetic wound treatments with various preparations on biomarkers of oxidative stress. (**A**): Nuclear factor erythroid 2-related factor-2 (Nrf-2); (**B**): malondialdehyde (MDA); (**C**): reduced glutathione (GSH). ###: Significantly different compared with the normal control group at *p* < 0.001, #### at *p* < 0.0001. *: Significantly different compared with DM + B-NEG group at *p* < 0.05, ** at *p* < 0.01, *** at *p* < 0.001, **** at *p* < 0.0001. $$$$: Significantly different compared with the CUR-G group at *p* < 0.0001. @: Significantly different compared with INS-G group at *p* < 0.05, @@ at *p* < 0.001.

As demonstrated in Figure 7, the levels of Nrf-2 and GSH were significantly depleted while the level of MDA was markedly increased in skin tissue homogenate from the DM + B-NEG group compared to the normal control group. Topical application of CUR-G, INS-G, and CUR-INS-NEG resulted in significantly higher levels of Nrf-2 and GSH and significantly lower levels of MDA compared with the DM + B-NEG group. Of note, the effect of CUR-INS-NEG was significantly higher than that of either CUR-G or INS-G. It is also interesting that the levels of MDA and GSH of the CUR-INS-NEG group were not significantly different from those in the normal group. Excessive ROS negatively impacts the healing process by triggering chronic inflammation, promoting pathogen expansion and infection, increasing apoptosis, disturbing the conventional sequence of the overlapping healing phases, and delaying tissue regeneration [93]. Several studies have reported that modulating oxidative stress is a promising approach to promote wound healing, particularly in diabetic wounds [94]. Hyperglycemia is a known trigger of oxidative stress due to the accumulation of advanced glycation end products in diabetic skin, inducing excessive ROS generation [95]. Compromised antioxidant defense mechanisms trigger ROS accumulation and lead to protein alteration, lipid peroxidation, and DNA damage, ultimately leading to accelerated cell death and retardation of wound healing [96]. Similarly, our results indicated an oxidant-antioxidant imbalance, as evidenced by increased MDA, a marker of lipid peroxidation, concomitant with reduced Nrf-2 and GSH levels. This imbalance was restored by the topical application of CUR-G, INS-G, and CUR-INS-NEG. Previous studies reported the antioxidant efficacy of CUR in diabetic wounds. CUR antibacterial dressings have been reported to enhance neovascularisation, collagen deposition, and re-epithelialization via their antioxidant, anti-inflammatory, and antibacterial effects [97]. Additionally, topical INS was reported to attenuate ROS levels in burn wounds [98].

#### 3.18.3. Effect of Treatments on Wound Inflammation

**Figure 8:**
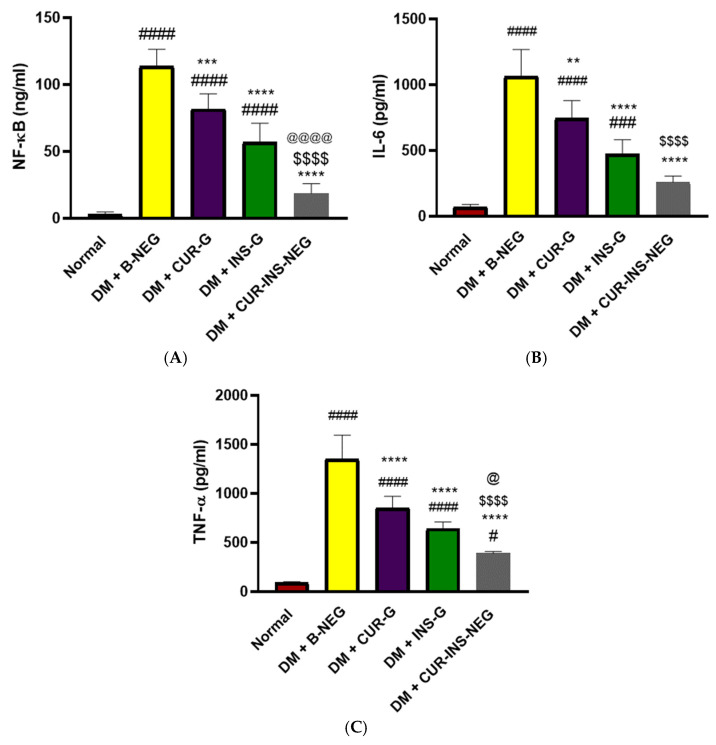
Effect of topical diabetic wound treatments with various preparations on skin biomarkers of inflammation. (**A**): Nuclear factor kappa-light-chain-enhancer of activated B cells (NF-κB); (**B**): tumor necrosis factor-α (TNF-α); (**C**): interleukin-6 (IL-6). #: Significantly different compared with the normal control group at *p* < 0.05, ### at *p* < 0.001, #### at *p* < 0.0001. **: Significantly different compared to DM + B-NEG group at *p* < 0.01, *** at *p* < 0.001, **** at *p* < 0.0001. $$$$: Significantly different compared to the CUR-G group at *p* < 0.0001. @: Significantly different compared to INS-G group at *p* < 0.05, @@@@ at *p* < 0.0001.

Figure 8 indicates increased levels of the inflammatory mediators NF-κB, IL-6, and TNF-α in skin tissue homogenate from the DM + B-NEG group compared with the normal group. Treatment with CUR-G, INS-G, or CUR-INS-NEG significantly reduced the levels of inflammatory mediators compared to the DM + B-NEG group. CUR-INS-NEG markedly demonstrated a significant reduction in all markers compared with CUR-G. In contrast, there was a significant difference in the levels of NF-κB and TNF-α only compared with INS-G. Interestingly, the levels of NF-κB and IL-6 in the CUR-INS-NEG group were comparable to those of the normal group. Persistent DM-associated oxidative stress triggers NF-κB-mediated inflammatory cytokine production, including TNF-α and interleukins, leading to chronic inflammation and retarded wound healing [99,100]. The NF-κB pathway plays a crucial role in delaying the diabetic wound-healing process, resulting in prolonged inflammation, impaired angiogenesis, and reduced cell proliferation. Moreover, NF-κB overexpression escalates cytokine synthesis, which promotes inflammation and oxidative stress in diabetic wounds [101]. Topical treatments with CUR-G, INS-G, and CUR-INS-NEG suppressed the high levels of inflammatory mediators in the diabetic wound area. Previous studies reported similar anti-inflammatory effects of CUR and INS nanoformulations in diabetic wounds [102,103].

#### 3.18.4. Effect of Treatment on Wound Histological Features

**Figure 9:**
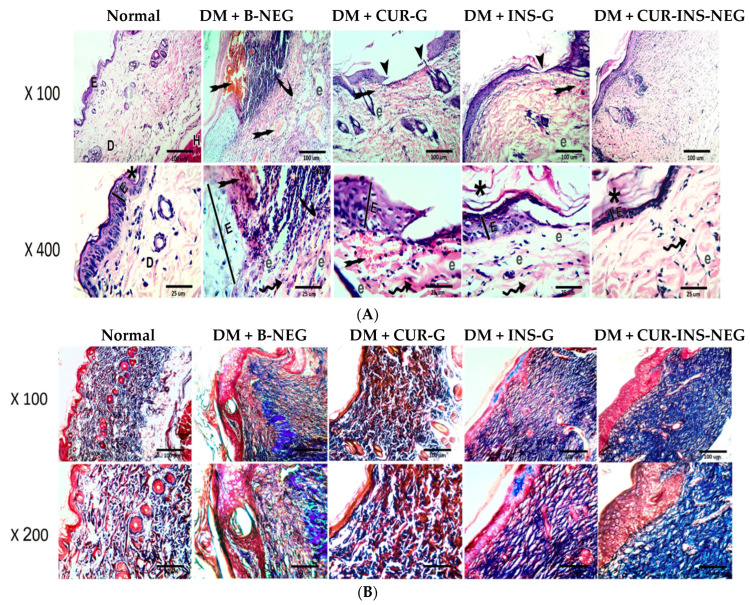
(**A**) Representative H&E photomicrograph of a section in the skin of different groups. 100×, bar = 100 µm, 400×, bar = 25 µm. (**B**) Representative Masson trichrome-stained sections showing deposition of collagen in the wound area from different experimental groups. The normal control group shows bluish-stained regular collagen bundles in the dermis separated by the skin appendages (hair follicles and sebaceous glands). The DM + B-NEG group shows loosely arranged, irregular collagen bundles and the absence of appendages in the dermis of the healed wound area underneath the thick, irregular epidermis. The DM + CUR-G group exhibits less pronounced irregular loose collagen bundle condensation in the dermis and a thinner epidermis compared to the DM + B-NEG group. The DM + INS-G group exhibits mildly regular, less condensed collagen surrounding hair follicles and sebaceous glands, with a thin, regular epidermis. The CUR-INS-NEG group shows regular, more condensed collagen bundles and a thin, regular overlying epidermis. The skin appendages appear to be normally presented. 100×, bar = 100 µm, 200×, bar = 50 µm.

Sections from the skin of the normal group show a normal histological appearance of the epidermis (E) with normal thickness (line) covered with thin keratin (star) and dermis (D) containing the skin appendages (hair follicles and sebaceous glands) overlying the hypodermis (H) (Figure 9A). Sections from the DM + B-NEG group show an interrupted thick epidermis (E) with extensive callus formation (curved arrow). Both the epidermis and dermis exhibit extensive vascular congestion (indicated by the tailed arrow) with numerous neutrophilic infiltrates. The dermal collagen bundles are irregular (double-curved arrow) and dispersed by obvious edema (e). The CUR-G-treated group shows a wide, interrupted, thick epidermis (E). The dermis is formed of irregular collagen bundles (double-curved arrow) and is dispersed by obvious edema (e) and extensive neutrophilic infiltrations with widespread congestion (tailed arrow). The INS-G-treated group shows minimal interrupted thickened epidermis (E) (arrowheads) covered with thick keratin (star). The dermis is formed of irregular collagen bundles (double-curved arrow) and dispersed by obvious edema (e) and less extensive neutrophilic infiltrations. The CUR-INS-NEG-treated group shows a non-interrupted thin epidermis (E) covered with keratin (star). The dermis is formed of more regular collagen bundles (double-curved arrow) with minimal interstitial edema (e) and scarce neutrophilic infiltrations.

#### 3.18.5. Effect of Treatment on Wound Collagen and TGF-β

**Figure 10:**
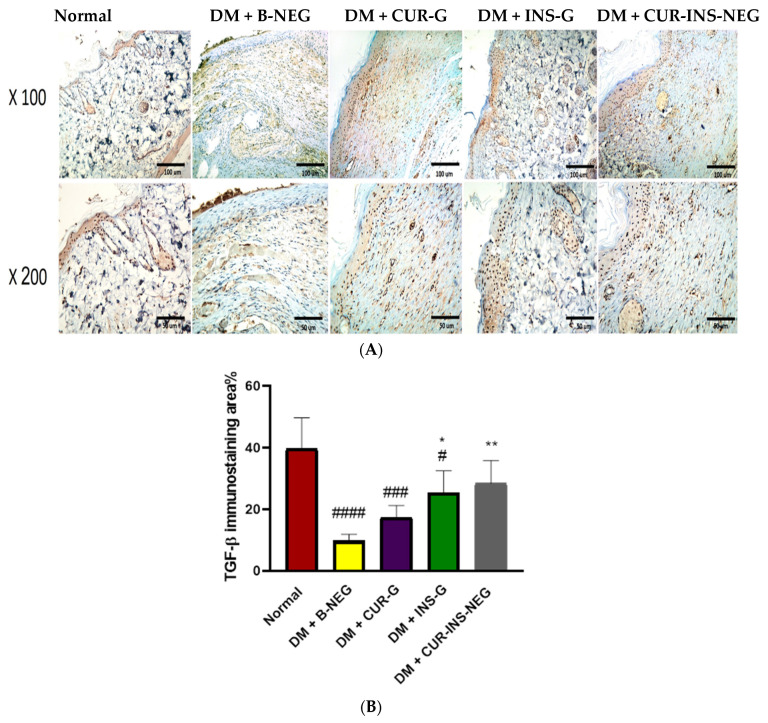
(**A**) Representative photomicrographs of TGF-β-immunostained skin section from different experimental groups, 100×, bar = 100 µm, 200×, bar = 50 µm. (**B**) Area % of TGF-β immunostaining in different experimental groups. #: Significantly different compared to the normal control group at *p* < 0.05, ### at *p* < 0.001, #### at *p* < 0.0001. *: Significantly different compared to the DM + B-NEG group at *p* < 0.05, ** at *p* < 0.01.

As shown in Figure 9B, sections from the DM + B-NEG group showed disorderly decreased collagen deposition. However, treatment with CUR-G, INS-G, and CUR-INS-NEG demonstrated restored collagen deposition in wound area tissue sections, with a superior effect of the CUR-INS-NEG treatment. Consistently, similar results were obtained for the levels of TGF-β as indicated in Figure 10. The DM + B-NEG group showed significantly reduced TGF-β immunostaining when compared to the normal control group. CUR-G, INS-G, and CUR-INS-NEG showed higher TGF-β levels compared with the DM + B-NEG group. However, the effects were only significant for the INS-G and CUR-INS-NEG groups (Figure 10B). Interestingly, TGF-β levels in the CUR-INS-NEG group were comparable to those of the normal group. Collagen is an extracellular matrix protein that promotes wound healing because it provides the mechanical strength required for tissue contraction and wound closure [104]. Impaired collagen deposition is a hallmark of diabetic wounds [105]. The topical CUR capacity to augment collagen deposition, facilitate granulation tissue production, and ultimately accelerate wound contraction has been confirmed in previous studies [106,107]. Additionally, INS positively affects wound healing through various mechanisms, including enhancement of re-epithelialization and deposition of extracellular matrix components, particularly collagen [108].

TGF-β is a cytokine that promotes wound healing by triggering collagen synthesis and granulation tissue formation [109]. DM inhibits fibroblasts’ ability to differentiate into myofibroblasts by altering the expression of many growth factors, particularly TGF-β [110]. TGF-β signaling pathway impairment prevents adaptive response for tissue repair. Therefore, restoring TGF-β expression is a promising strategy for enhancing wound healing. Thus, Rezaii et al. reported improved cutaneous wound healing by CUR nanoparticles topical application through the upregulation of TGF-β expression [111].

#### 3.18.6. Effect of Treatment on Wound VEGF

**Figure 11:**
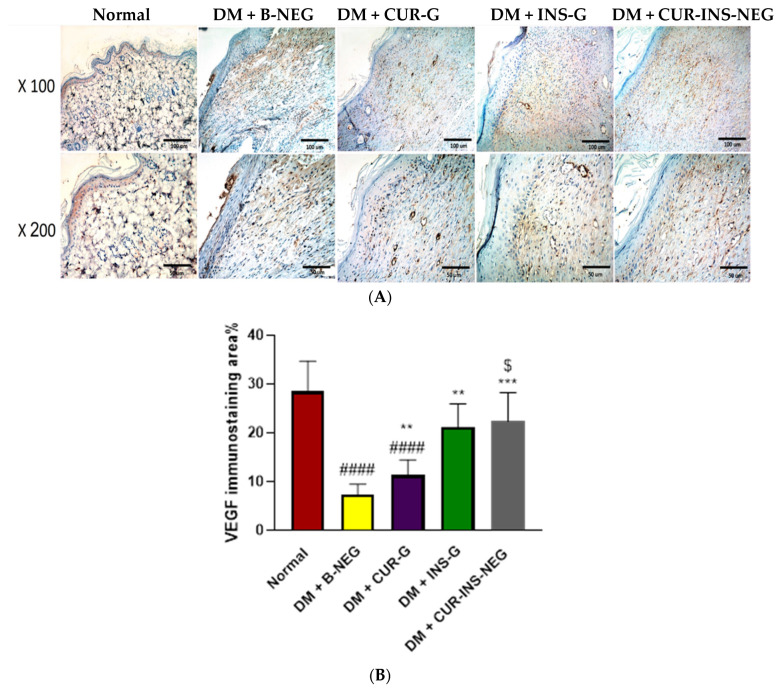
(**A**) Representative photomicrograph of VEGF-immunostained skin sections from different experimental groups, 100×, bar = 100 µm, 200×, bar = 50 µm. (**B**) Area % of VEGF immunostaining. ####: Significantly different compared to the normal control group at *p* < 0.0001. **: Significantly different compared to DM + B-NEG group at *p* < 0.01, *** at *p* < 0.001. $: Significantly different compared to CUR-G-treated group at *p* < 0.05.

Figure 11 demonstrates significantly decreased VEGF expression in the DM + B-NEG group in comparison with the normal control group. In contrast, different treatment groups demonstrated an increase in VEGF expression, which was significantly different in all the treated groups compared with the DM + B-NEG group. Additionally, CUR-INS-NEG showed significantly higher VEGF levels compared with the CUR-G group. Moreover, the levels in the INS-G and CUR-INS-NEG groups were comparable to those in the normal group (non-significant difference). Angiogenesis is essential to maintain the newly formed granulation tissue. It is mainly promoted by growth factors such as TGF-β and VEGF [16]. VEGF promotes the proliferation of endothelial cells and prevents their apoptosis, while TGF-β promotes angiogenesis by enhancing VEGF expression or independently by stimulating new blood vessel formation. CUR has been reported to up-regulate VEGF and TGF-β in diabetic and non-diabetic wounds [109,112,113]. Similarly, topical application of INS gel increased the expression of VEGF and VEGF receptor I in wounds of hyperglycemic mice [114].

These mechanistic in vivo studies in a DM wound model clearly demonstrate the superior performance of the three tested preparations compared with the groups treated with the blank nanoemulgel. It is noted that INS-G outperformed CUR-G in all the tested measurements, which is likely attributed to the high binding affinity of INS to its receptors and IR/IGF-1R hybrid receptors, directly triggering potent pro-regenerative signaling, unlike CUR, which has no direct receptor-binding effects [115,116]. The impact of CUR-INS-NEG was notably higher than that of either CUR-G or INS-G in all the measured parameters, which is likely attributed to several factors. The nanometric size has been found to increase drug penetration through the skin and improve drug wound-healing efficacy [9,29,33,117,118]. PL-F127 and CS were also found to improve the residence time at the wound sites through their mucoadhesive properties, leading to improved efficacy [41,119]. Lastly, previous studies suggested that the combination of CUR and INS might offer better effects compared to either drug alone, through synergistic and additive effects [92].

Although this study included free INS and free CUR groups, as well as a blank NEG containing myrrh oil to account for vehicle-related effects, the effect of non-formulated (free) myrrh oil was not independently evaluated, which is a limitation of this study. Therefore, while active component and formulation effects were differentiated, the specific contribution of myrrh oil outside the NE system remains to be further clarified. Additionally, inclusion of a non-diabetic wounded group could have provided a more comprehensive comparison under normoglycemic conditions. The potential synergistic effects between the NEG ingredients were not formally evaluated. Future studies are warranted to further delineate these aspects and to formally evaluate potential synergistic effects using the Chou–Talalay method and isobolographic analysis.

## 4. Conclusions

A CUR-INS-NEG system containing myrrh oil, CUR, and INS was successfully developed for the potential management of diabetic wounds. This NEG formulation combines the anti-inflammatory, antioxidant, and wound-healing properties of its components, taking advantage of their potential complementary effects. CUR NE formulations were optimized by employing a three-factor two-level D-optimal mixture design to study the independent variable effects on the responses. The Smix content of the NE had the strongest effect on the NE droplet size, PDI, zeta potential, and drug content%. This suggests the possibility of fine-tuning these properties by careful NE composition adjustment. FT-IR and DSC studies confirmed the absence of interactions among the NE ingredients. A PL-F127-CS mixture was used to formulate the NEG to take advantage of its thermoreversible and mucoadhesive properties. CUR-INS-NEG showed favorable properties, including pH, viscosity, spreadability, and sustained drug release, which confirm its potential clinical benefits. This formulation also demonstrated improved in vitro antibacterial activity compared to its individual components, as confirmed by higher inhibition zones and anti-biofilm activity, and lower MICs. Moreover, it demonstrated better in vivo wound-healing effects compared with B-NEG, INS-G, and CUR-G. Detailed mechanistic studies indicated that this improvement was due to enhanced anti-inflammatory and antioxidant properties. Additionally, CUR-INS-NEG showed increased collagen deposition, granulation tissue formation, and endothelial cell proliferation. These findings confirm the potential of CUR-INS-NEG for the effective management of diabetic wounds by integrating the therapeutic benefits of myrrh oil, CUR, and INS with the enhanced delivery performance of NEG.

## Figures and Tables

**Table 1:** Independent and dependent variables of CUR-loaded NEs.

Independent Variables (% *v*/*v*)	Level of Variables		
	Range	Low	High
X_1_ = Oil%	10–15	10	15
X_2_ = Smix%	40–50	40	50
X_3_ = Water%	40–50	40	50
Dependent variables	Goal		
Y_1_ = Particle size (nm)	Minimize		
Y_2_ = PDI	Minimize		
Y_3_ = Zeta potential (mV)	Maximize		
Y_4_ = Drug content (%)	Maximize		

**Table 2:** Independent variables and the obtained responses of various CUR-loaded NE formulations.

Independent Variables				Responses			
	Oil (%) (X_1_)	Smix (%)(X_2_)	Water (%) (X_3_)	Particle Size (nm) (Y_1_)	PDI (Y_2_)	Zeta Potential (mV) (Y_3_)	Drug Content % (Y_4_)
NE1	11	48	41	164.1 ± 0.8	0.29 ± 0.02	−31.1 ± 0.9	97.9 ± 0.1
NE2	10	40	50	308.2 ± 2.9	0.35 ± 0.01	−23.8 ± 0.3	95.0 ± 0.3
NE3	15	44	41	276.0 ± 6.5	0.34 ± 0.01	−24.9 ± 1.2	95.3 ± 0.7
NE4	12	40	48	326.4 ± 1.3	0.32 ± 0.03	−22.9 ± 0.6	94.3 ± 0.4
NE5	13	43	44	251.2 ± 1.6	0.34 ± 0.01	−26.6 ± 1.5	95.4 ± 0.4
NE6	10	45	45	194.4 ± 4.8	0.30 ± 0.02	−28.2 ± 0.4	96.1 ± 0.1
NE7	13	47	40	193.8 ± 3.4	0.30 ± 0.03	−29.8 ± 2.3	96.8 ± 0.3
NE8	10	43	47	246.2 ± 5.2	0.32 ± 0.01	−27.0 ± 0.1	95.8 ± 0.4
NE9	10	47	43	166.0 ± 2.1	0.30 ± 0.02	−30.4 ± 1.4	97.0 ± 0.1
NE10	12	45	42	221.0 ± 2.4	0.31 ± 0.03	−27.8 ± 0.3	96.0 ± 0.8
NE11	15	40	45	349.7 ± 10.9	0.30 ± 0.01	−21.9 ± 0.7	93.0 ± 0.6
NE12	10	50	40	155.2 ± 0.8	0.28 ± 0.00	−31.4 ± 0.8	98.3 ± 0.6

**Table 3:** Properties of various gel preparations.

Formulation	pH	Gelation Temperature (°C)	Spreadability (mm)	Viscosity (P)
Control PL-F127	6.94 ± 0.03	35.70 ± 0.42	60.81 ± 0.80	122 ± 1.73
CS-PL-F127	6.22 ± 0.07	34.70 ± 0.15	41.12 ± 0.3	226 ± 6.24
B-NEG	6.55 ± 0.07	35.13 ±0.05	51.30 ± 0.55	182 ± 4.58
CUR-G	6.26 ± 0.04	34.96 ± 0.11	55.70 ± 0.45	166 ± 4.58
INS-G	6.15 ± 0.03	34.63 ± 0.15	57.20 ± 0.36	156 ± 3.0
CUR-INS-NEG	6.01 ± 0.09	34.20 ± 0.21	47.90 ± 0.60	219 ± 9.0

**Table 4:** Storage stability of CUR-INS-NEG.

Parameter	Freshly Prepared	Stored for 90 Days
Color	Transparent yellow	Transparent yellow
Homogeneity	Homogenous	Homogenous
pH	6.01 ± 0.09	5.95 ± 0.13
Viscosity (P)	219 ± 9.0	289 ± 1.73
INS content (%)	98.8 ± 0.36	94.8 ± 0.31
Droplet size (nm)	20.70 ± 1.00	21.01 ± 0.69
PDI	0.29 ± 0.005	0.32 ± 0.007
Zeta potential (mV)	46.66 ± 3.72	41.66 ± 0.11

## Data Availability

The data presented in this study are available on request from the corresponding author.

## Supplementary Materials

The following supporting information can be downloaded at: https://www.mdpi.com/article/10.3390/pharmaceutics18030369/s1, Table S1. Model selection for droplet size, PDI, zeta potential, and drug content% using D-optimal mixture design. Table S2. ANOVA for the models used for droplet size (Y1), PDI (Y2), ZP (Y3), and drug content% (Y4). Figure S1. Optimization plot showing the selected NE composition and corresponding response outcomes.

## Abbreviations

The following abbreviations are used in this manuscript:

CUR	Curcumin
NEs	Nanoemulsions
INS	Insulin
NEG	Nanoemulgel
DM	Diabetes mellitus
PL-F127	Pluronic^®^ F127
TW80	Tween 80
CS	Chitosan
TEM	Transmission electron microscopy
FT-IR	Fourier transform infrared
DSC	Differential scanning calorimetry
MICs	Minimum inhibitory concentrations
STZ	Streptozotocin
H&E	Hematoxylin and eosin
ELISA	Enzyme-linked immunosorbent assay
Nrf-2	Nuclear factor erythroid 2-related factor 2
MDA	Malondialdehyde
GSH	Reduced glutathione
NF-κB	Nuclear factor kappa-light-chain-enhancer of activated B cells
TNF-α	Tumor necrosis factor alpha
IL-6	Interleukin-6
TGF-β	Transforming growth factor-β
VEGF	Vascular endothelial growth factor
ZP	Zeta potential
ROS	Reactive oxygen species
