# Supplementary material for: Myrrh Oil-Based Nanoemulsion Loaded with Curcumin and Insulin: Development, Characterization, and Evaluation of Enhanced Antibacterial and Diabetic Wound-Healing Activity
Source: Pharmaceutics. 2026 Mar 16;18(3):369. doi: 10.3390/pharmaceutics18030369 (PMC13030445; doi:10.3390/pharmaceutics18030369)
Supplement: Supplementary file 1 [file pharmaceutics-18-00369-s001.zip › pharmaceutics-4162512-supplementary.pdf]

Supplementary information for:

Myrrh oil-based nanoemulsion loaded with curcumin and insulin: Development, characterization, and evaluation of enhanced antibacterial and diabetic wound-healing activity

Table S1. Model selection for droplet size, PDI, zeta potential, and drug content% using D-optimal mixture design.

Response 1: Droplet size (Y_1)				
Source	Sequential *p*-value	Adjusted R^2	Predicted R^2	
Linear	< 0.0001	0.9437	0.9017	
Quadratic	0.0032	0.9902	0.9689	Suggested
Special Cubic	0.1879	0.9920	0.9664	
Cubic	0.8917	0.9846	-37.3925	
Sp Quartic vs Quadratic	0.2750	0.9938	-47.7649	
Quartic vs Cubic				Aliased
Quartic vs Sp Quartic				Aliased
Response 2: PDI (Y_2)				
Source	Sequential *p*-value	Adjusted R^2	Predicted R^2	
Linear	0.0462	0.3830	-0.1782	
Quadratic	0.1788	0.5681	-0.6680	
Special Cubic	0.0330	0.8083	0.2816	Suggested
Cubic	0.4177	0.8550	-352.2499	
Sp Quartic vs Quadratic	0.0268	0.9449	-343.2724	Suggested
Quartic vs Cubic				Aliased
Quartic vs Sp Quartic				Aliased
Response 3: ZP (Y_3)				
Source	Sequential *p*-value	Adjusted R^2	Predicted R^2	
Linear	< 0.0001	0.9440	0.9071	Suggested
Quadratic	0.1238	0.9657	0.8955	
Special Cubic	0.3565	0.9659	0.8880	
Cubic	0.8325	0.9406	-78.0670	
Sp Quartic vs Quadratic	0.4072	0.9707	-143.9732	
Quartic vs Cubic				Aliased
Quartic vs Sp Quartic				Aliased
Response 4: drug content% (Y_4)				

Source	Sequential *p*-value	Adjusted R^2	Predicted R^2	
Linear	< 0.0001	0.9375	0.8951	Suggested
Quadratic	0.1147	0.9628	0.9096	
Special Cubic	0.4676	0.9603	0.8567	
Cubic	0.4448	0.9678	-44.2637	
Sp Quartic vs Quadratic	0.8014	0.9444	-572.3122	
Quartic vs Cubic				Aliased
Quartic vs Sp Quartic				Aliased

Table S2. ANOVA for the models used for droplet size (Y_1), PDI (Y_2), ZP (Y_3), and drug content% (Y_4).

Response 1: Droplet size (Y_1)						
Source	Sum of Squares	df	Mean Square	F-value	p-value	
Model	48278.75	5	9655.75	224.26	< 0.0001	significant
(1)Linear Mixture	46300.86	2	23150.43	537.68	< 0.0001	
AB	429.57	1	429.57	9.98	0.0196	
AC	104.07	1	104.07	2.42	0.1710	
BC	1339.94	1	1339.94	31.12	0.0014	
Residual	258.34	6	43.06			
Cor Total	48537.09	11				
R square	0.9947					
C.V.%	2.76					
Adeq Precision	43.8524					
Response 2: PDI (Y_2)						
Source	Sum of Squares	df	Mean Square	F-value	p-value	
Model	0.0045	6	0.0008	8.73	0.0155	significant
(1)Linear Mixture	0.0025	2	0.0012	14.21	0.0087	
AB	6.803E-07	1	6.803E-07	0.0078	0.9328	
AC	0.0002	1	0.0002	2.73	0.1594	
BC	0.0001	1	0.0001	0.7539	0.4249	
ABC	0.0007	1	0.0007	8.52	0.0330	
Residual	0.0004	5	0.0001			
Cor Total	0.0050	11				
R square	0.9129					
C.V.%	3					
Adeq Precision	10.0925					
Response 3: ZP (Y_3)						
Source	Sum of Squares	df	Mean Square	F-value	p-value	
Model	110.14	2	55.07	93.74	< 0.0001	significant
(1)Linear Mixture	110.14	2	55.07	93.74	< 0.0001	
Residual	5.29	9	0.5875			

Cor Total	115.43	11				
R square	0.9542					
C.V.%	2.82					
Adeq Precision	27.1245					
Response 4: drug content% (Y_4)						
Source	Sum of Squares	df	Mean Square	F-value	p-value	
Model	22.81	2	11.41	83.56	< 0.0001	significant
(1)Linear Mixture	22.81	2	11.41	83.56	< 0.0001	
Residual	1.23	9	0.1365			
Cor Total	24.04	11				
R square	0.9489					
C.V.%	0.3852					
Adeq Precision	26.1131					

(1) Inference for linear mixtures uses Type I sums of squares.
A is the oil concentration(%), B is the Smix concentration (%) and C is the water concentration (%).

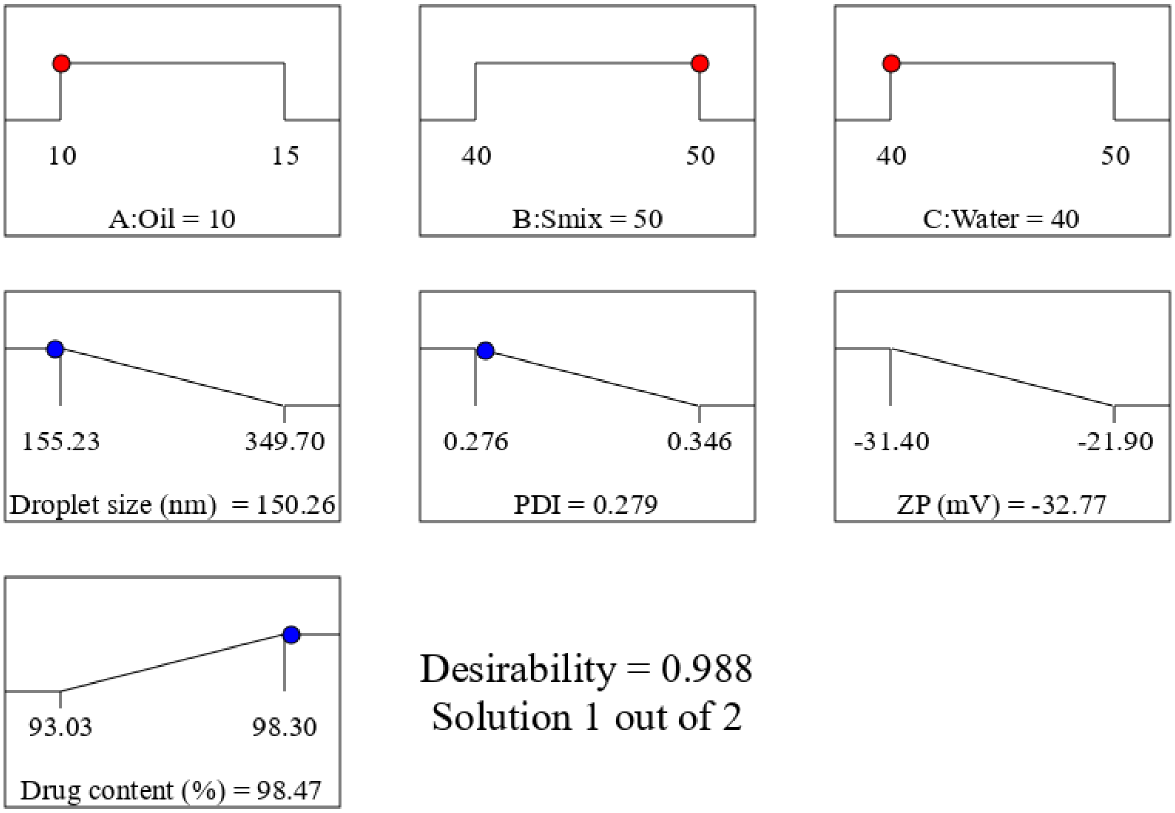

Figure S1. Optimization plot showing the selected NE composition and corresponding response outcomes.
